# Uncooled High Detectivity Mid-Infrared Photoconductor Using HgTe Quantum Dots and Nanoantennas

**DOI:** 10.1021/acsnano.3c12581

**Published:** 2024-03-11

**Authors:** Augustin Caillas, Philippe Guyot-Sionnest

**Affiliations:** James Franck Institute, The University of Chicago, 929 East 57th Street, Chicago, Illinois 60637, United States

**Keywords:** colloidal quantum dots, nanoantennas, infrared photodetection, photoconductor, specific detectivity

## Abstract

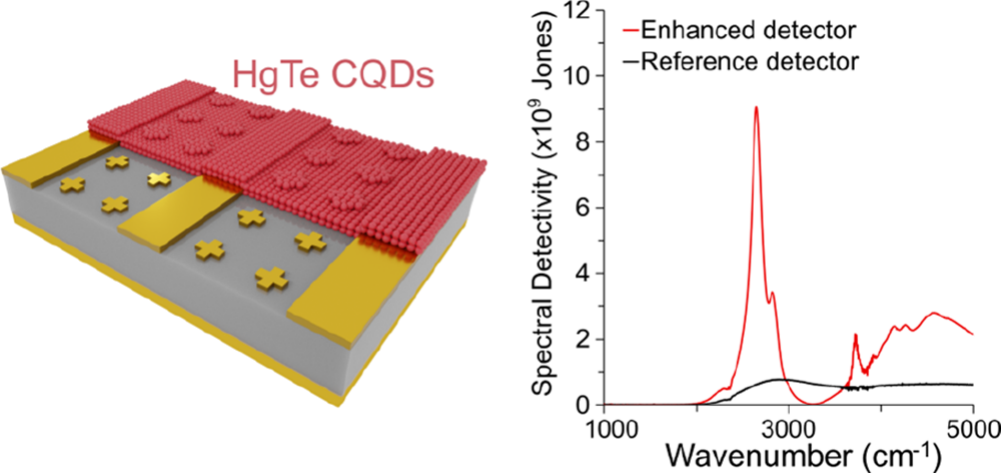

Using a metal/insulator/metal (MIM) structure with a gold nanoantenna array made by electron beam lithography, the responsivity of a HgTe colloidal quantum dot film is enhanced in the mid-infrared. Simulations indicate that the spatially averaged peak spectral absorption of an 80 nm film is 60%, enhanced 23-fold compared to that of the same film on a bare sapphire substrate. The field intensity enhancement is focused near the antenna tips, being 20-fold 100 nm away, which represents only 1% of the total area and up to 1000-fold at the tips. The simulated polarized absorption spectra are in good agreement with the experiments, with a strong resonance around 4 μm. A responsivity of 0.6 A/W is obtained at a 1 V bias. Noise measurements separate the 1/f noise from the generation–recombination white noise and give a spatially averaged photoconductive gain of 0.3 at 1 V bias. The spatially averaged peak detectivity is improved 15-fold compared to the same film on a sapphire substrate without an MIM structure. The experimental peak detectivity reaches 9 × 10^9^ Jones at 2650 cm^–1^ and 80 kHz, decreasing at lower frequencies. The MIM structure also enhances the spatially averaged peak photoluminescence of the CQD film by 16-fold, which is a potential Purcell enhancement. The good agreement between simulations and measurements confirms the viability of lithographically designed nanoantenna structures for vastly improving the performance of mid-IR colloidal quantum dot photoconductors. Further improvements will be possible by matching the optically enhanced and current collection areas.

Over the past decade, colloidal quantum dots (CQDs) have demonstrated a growing potential for mid-infrared photodetection.^1^ In particular, HgTe CQDs have emerged as a major candidate to provide an alternative to epitaxial materials for photodetection and imaging in the infrared (IR).^2,3^ It is expected that further progress of HgTe CQD photodetector devices will rely equally on engineering the material properties^4−6^ and the photodetector architectures. Improved architecture can focus on increasing the light absorption with the minimum amount of material in order to increase light collection efficiency and minimize noise. Examples of this strategy include plasmonic antennas,^7−9^ guided mode resonance,^10^ Helmholtz resonators,^11^ plasmonic gratings,^12^ optical cavities,^13,14^ and distributed Bragg mirrors.^15^ Lateral and vertical devices have been explored. Photoconductor devices have so far displayed lower detectivity than photovoltaic devices because of the inherent 1/f noise associated with the dark current. At present in the mid-infrared, the highest reported room temperature detectivity has been 7.6 × 10^9^ at 2815 cm^–1^,^16^ and 1.1 × 10^9^ at 2675 cm^–1^.^17^ The detectivity values can be compared to those of commercial thin film lithium tantalate pyroelectric sensors for which D* is about 3 × 10^8^ Jones at sub 100 Hz bandwidth.^18^ These low-cost but slow detectors are widely used for optical gas sensing along with modulated thermal light sources. Gas sensors could then advantageously use quantum dot detector devices with a much higher detectivity. Coupled with the increasing availability of fast modulated mid-IR light emitting diodes, the 1/f noise of photoconductor devices could be mitigated. Reducing the detector bandwidth to the absorption bands of interest could also be a benefit.

The long-term goal of this work is to use Purcell enhancement to increase the radiative recombination efficiency of mid-infrared CQDs in order to bring the detectivity closer to the background limit at room temperature.^19^ This article focuses on optical enhancement for a room temperature mid-IR photoconductor based on HgTe CQDs using metallic nanoantennas in a metal/insulator/metal (MIM) structure. It combines optical simulations and experiments to explore the spatially averaged responsivity and detectivity for ∼100 nm thin films of HgTe colloidal quantum dot photoconductors in the mid-infrared. The MIM structure should also enhance the luminescence of the CQD film if it does not simultaneously enhance nonradiative processes, and this is tested by photoluminescence (PL) measurements.

## Results and Discussion

### Design of the Photodetector

**Figure 1:**
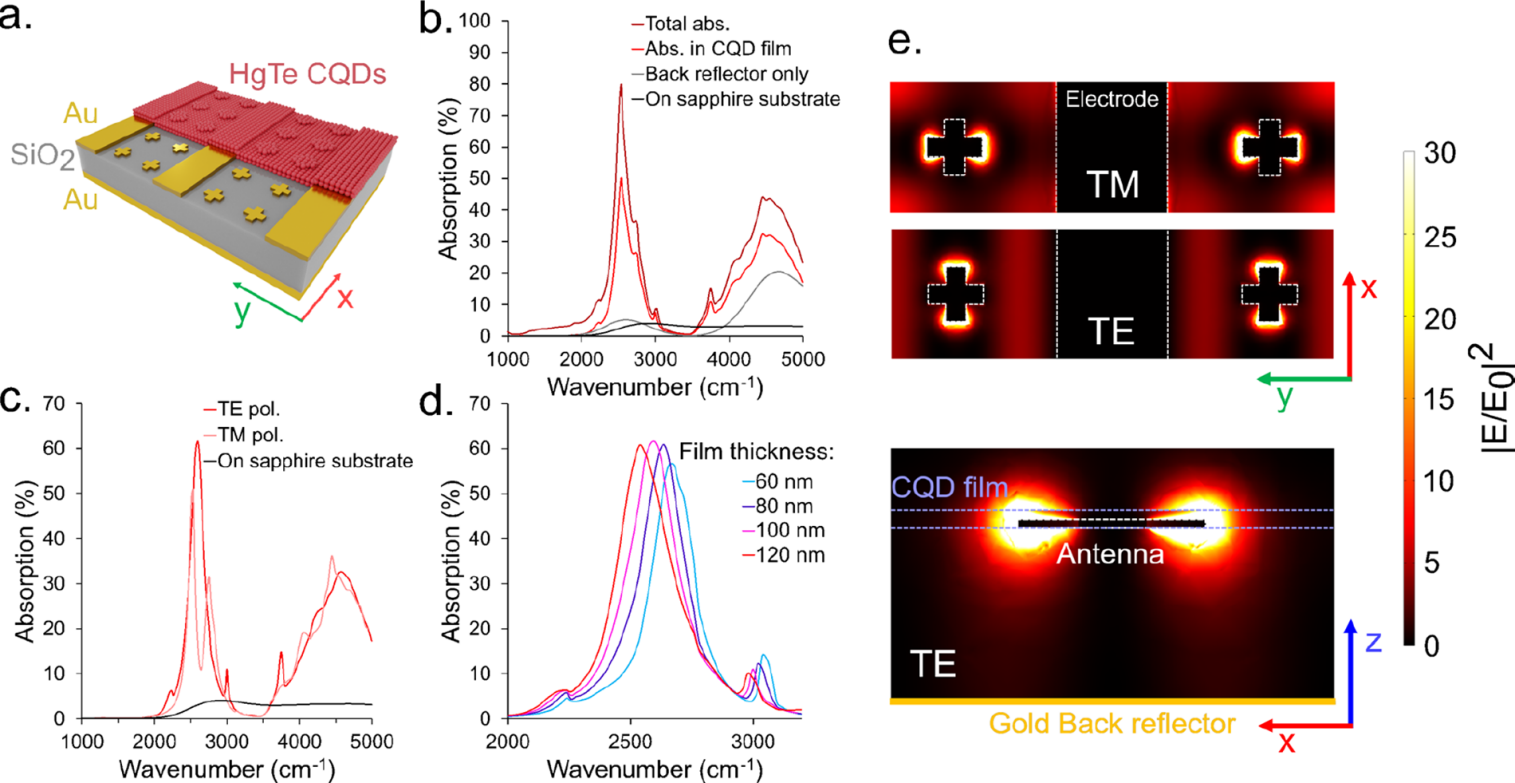
(a) Schematic of the MIM photodetector design. (b) Simulated absorption in the entire MIM structure (dark red), in the HgTe CQD layer of the MIM structure, in the same CQD film but deposited on a substrate lacking nanoantennas, and in the same CQD film but deposited on a bare sapphire substrate. (c) Simulated absorption in the HgTe CQD layer of an MIM structure for TE polarized illumination (red) and TM polarized illumination (light red) and simulated absorption in the same HgTe CQD layer deposited on a sapphire substrate (black). (d) Simulated absorption in the HgTe CQD layer of a MIM structure determined as a function of the HgTe CQD layer thickness. (e) Field maps of the electromagnetic field enhancement from a top view for TE and TM polarized illumination and from a side view for TE polarized illumination. The maximum intensity is concentrated close to the vicinity of the cross antennas.

The device relies on an MIM resonant structure to enhance the optical absorption in a thin film of HgTe CQD within a narrow spectral band. The architecture is depicted in Figure 1a. The bottom layer of the structure consists of a 1000 nm thick SiO_2_ layer deposited on top of a gold back reflector. This behaves as a quarter wave layer with a relatively broad first order resonance at 1800 cm^–1^ and a third order resonance at 5300 cm^–1^. On top, there is an array of periodic gold nanoantennas placed within the conductive channels formed by interdigitated electrodes. Similar designs have been widely used as perfect absorbers,^20−24^ for absorption enhancement in photodetectors,^25−27^ and for SERS and SEIRA applications.^28,29^ In particular, a similar architecture for a PbS quantum dot photoconductor in the near-infrared has been recently demonstrated.^26^ To turn the MIM structure into a photodetector, a layer of mid-IR HgTe CQDs is deposited directly on top of the electrodes and metallic antennas.

### Simulations

Finite element simulations were conducted to maximize the absorption of the CQD layer in the mid-IR, for a 4 μm wavelength. The antenna array is composed of gold crosses with an arm length of 1100 nm and width of 400 nm and with a 2600 nm periodicity. Two periods of the array can fit in the 6700 nm wide conductive channel between the 2200 nm wide gold electrodes. The electrodes and antennas are 50 nm thick. With a 100 nm thick CQD top layer, the total absorption of the structure (gold and CQD) reaches 80% at the top of a narrow peak at 2550 cm^–1^ for unpolarized illumination at normal incidence (Figure 1b). This peak originates from the combination of the quarter-wave resonance from the bottom layers and the plasmonic resonance from the gold nanoantennas, as supported by the simulated distribution of the electric field in the structure (Figure 1e). The broader absorption peak at ∼4600 cm^–1^ is associated with the  resonance of the bottom layers, while the absorption is minimized at 3300 cm^–1^ as a result of the  destructive interferences.

To optimize the structure for photodetection, it is necessary to maximize the absorption in the CQD film. It is calculated by integrating the dissipated electromagnetic power density over the volume of the CQD layer, and it reaches 50%. We note that the maximum absorption in the CQD film is obtained when the peak of the quarter wave resonance and the peak of the plasmonic resonance are spectrally displaced from each other as this results in reduced losses in the nanoantennas (Figure S1).

Compared to the absorption of a 100 nm thick CQD layer on top of a sapphire transparent substrate (Figure 1b), the MIM architecture provides an enhancement of the absorption by a factor of 19 at the peak wavelength. Figure 1c shows that this enhancement depends on polarization, as the structure displays higher absorption for an incident field polarized along the electrodes (TE) than perpendicular to the electrodes (TM). The polarization dependence is due to the interdigitated electrodes that break the symmetry of the nanoantenna array in the direction perpendicular to their length, effectively mitigating the resonance for TM polarization. At non-normal incidence the absorption for TM polarization decreases further, while it remains relatively unchanged for TE polarization for angles less than 5° (Figure S2), making TE polarization suitable for photodetection with a F/5 focusing optic. For comparison, simulations were also conducted for a structure featuring only a quarter wave resonance without the antennas in the conductive channel (Figure S3). In this case the enhancement of the CQD layer absorption at normal incidence is 5-fold and it is spatially uniform.

The field maps in the MIM structure for TE and TM polarization (Figure 1e) show that the vast majority of the absorption takes place in the vicinity of the nanoantennas. The field intensity enhancement  reaches 1000 within a few nanometers of the antenna tips and remains larger than 20, 100 nm away from the tips. Therefore, most of the absorption occurs within a very small fraction of the film. The field is also strongly confined above the antennas so that a high absorption can be obtained with a thin film of CQDs. Figure 1d shows the absorption spectra for different film thicknesses.

The concentration of the optical absorption within a small volume is useful since the thermal noise of a photodetector increases with the volume of the active material. As shown in Figure 1b, this strong spatial confinement of the resonance in the vicinity of the antennas is obtained at the expense of about 30% optical losses in the metal structures. The simulation gives a maximum absorption enhancement in the CQD film for an 80 nm thick film with an enhancement by a factor of 23 and absorption reaching 60% for TE polarized illumination. Increasing the CQD film thickness to several hundreds of nanometers mitigates the MIM resonance and results in much lower absorption enhancement factors (Figure S4).

### MIM Photodetector Characterization

**Figure 2:**
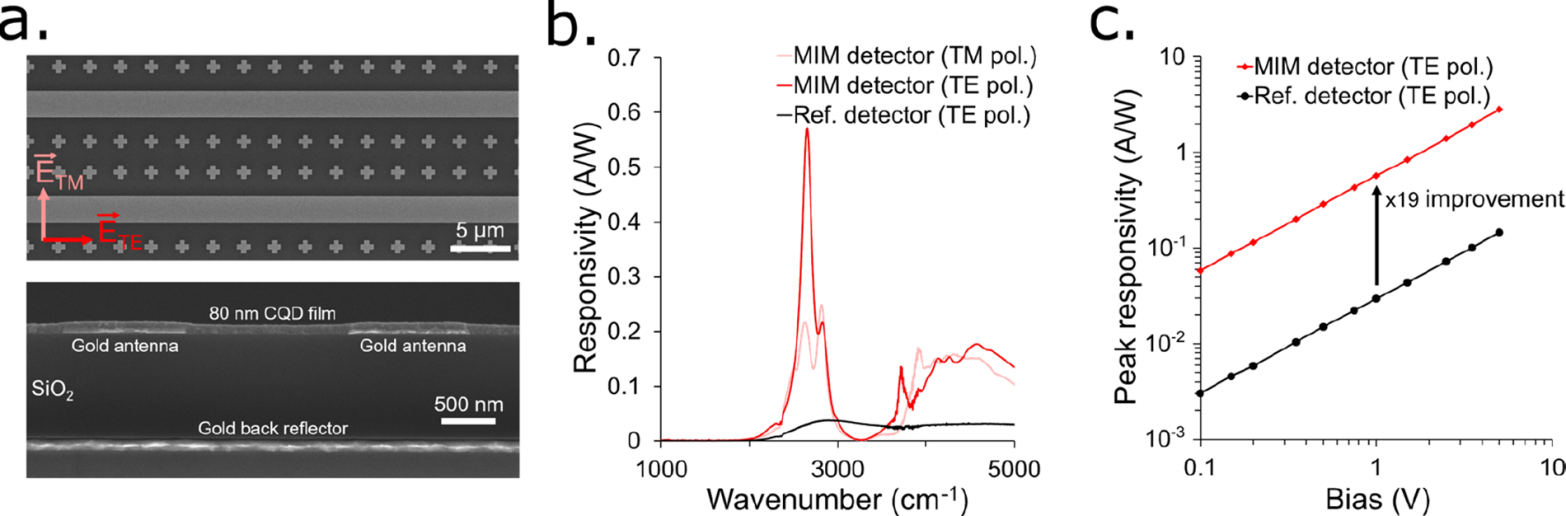
(a) SEM images of the MIM photodetector. Top: top view. Bottom: a slice of the device. (b) Responsivity spectrum of the MIM photodetector for illumination with TM polarization (light red) and TE polarization (red) and for the reference photodetector with TE polarized illumination (black). (c) Responsivity at 2650 cm^–1^ for the MIM photodetector (red) and the reference detector (black) for TE polarized illumination.

Figure 2a shows SEM images of the top surface and the cross-section of the MIM photodetector. The antennas and electrodes were made during the same e-beam lithography session in order to obtain the best alignment. On the same substrate, reference photodetectors were fabricated with the same electrodes but without nanoantennas and back reflectors. All devices were patterned within a small area of the substrate to reduce the influence of film thickness variation on the measured responsivity. The detectors comprise 59 channels of 6.7 × 500 μm, making each device active area 0.198 mm^2^. The total surface area covered by the device is 0.261 mm^2^. An 80 nm thick CQD film was deposited by performing two spin coatings of the CQD solution and treating each layer with ethanedithiol (EDT). The resulting film was then treated with a solution of sulfur ions in octylamine to reduce the inherent n-doping of the film in order to reduce the electrical noise of the detector. The doping was then further reduced by baking the device in air at 60 °C for varying times.

The MIM photodetector displays a linear IV curve (Figure S5). The experimental spectral responsivity (Figure 2b) matches rather well the predicted resonance characteristic of the MIM structure (Figure 1c). The TE responsivity spectrum displays a narrow peak at 2650 cm^–1^ as predicted. The TM spectrum shows two weaker resonances at around 2650 cm^–1^. The MIM detector spectra are compared to the reference detector. This one also shows a polarization-dependent responsivity because the electrodes act as a weak diffraction grating, slightly reducing the absorption for TM polarized illumination (Figure S6). For TE polarization, at the peak wavelength, the responsivity of the MIM detector is enhanced 19-fold over the reference detector, which is in good agreement with the simulated 23-fold absorption enhancement. The slightly lower enhancement is explained by the illumination of the device at non-normal incidence with F/5 optics and may also be due to the increased losses in the fabricated nanoantennas due to their finite thickness, edge and surface roughness, as well as other possible defects such as broken or damaged nanoantennas, asymmetric nanoantennas, and size and thickness imperfections. We find that the quality of the agreement is rather striking, and that it bodes very well for further use of simulations and fabrication at these length scales.

Figure 2c shows the responsivity of the MIM detector and the reference detector for TE polarized illumination at the MIM structure’s resonant wavelength (2650 cm^–1^) as a function of bias. The responsivity increases linearly with bias for both devices, with a constant 19-fold enhancement. The responsivity of the MIM detector reaches more than 1 A/W for biases larger than 2.5 V, thanks to the large absorption enhancement provided by the resonant structure.

**Figure 3:**
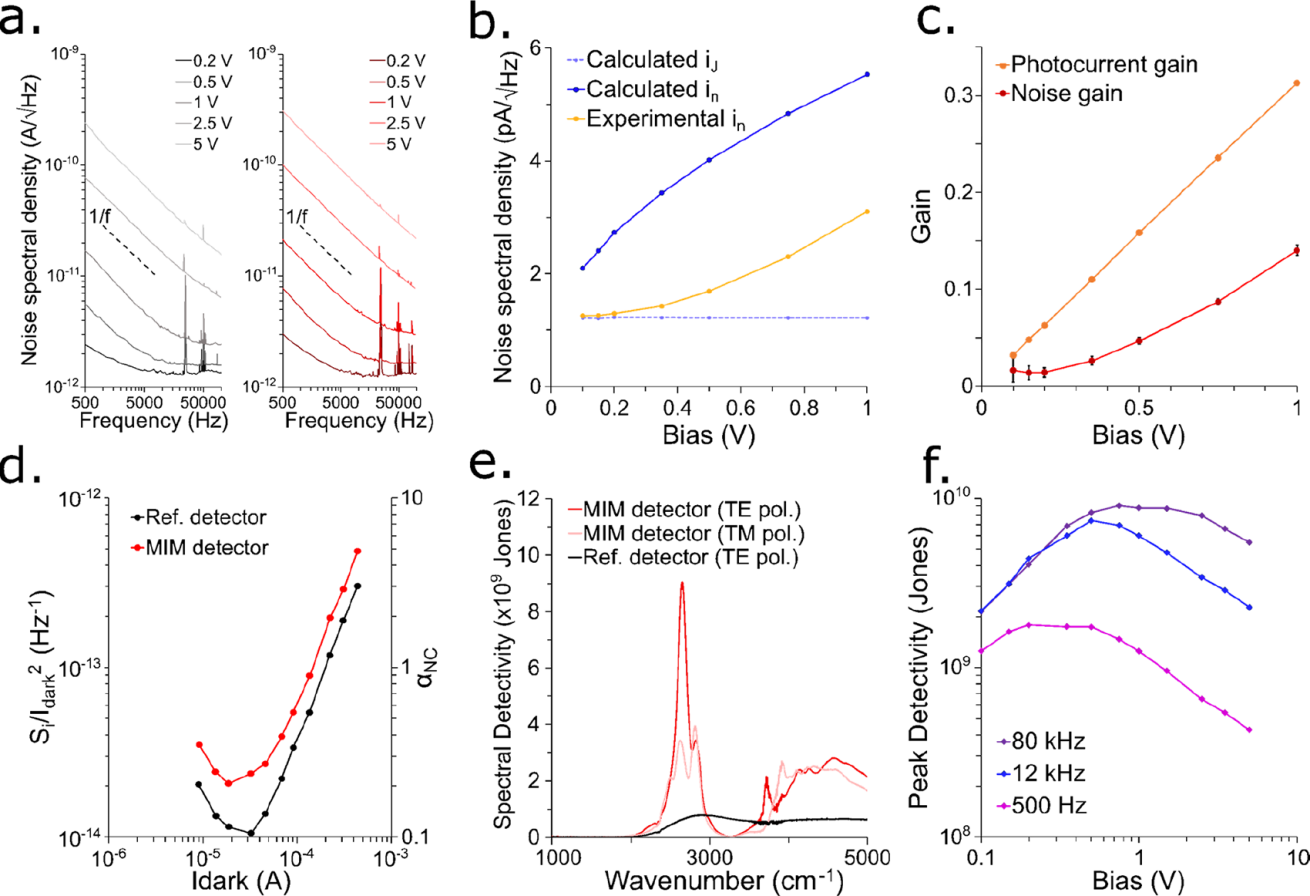
(a) Noise spectral density spectra for increasing bias for the reference photodetector (left) and the MIM photodetector (right). (b) Experimental white noise and calculated white noise as a function of bias for the MIM photodetector. (c) Extracted values of *G*_ph_ and *G*_noise_. (d) 1/f noise of the reference photodetector and MIM photodetector at 500 Hz. (e) Room temperature spectral specific detectivity of the MIM photodetector under TE and TM polarized illumination at 0.75 V and at 80 kHz. (f) Room temperature spectral detectivity at 2650 cm^–1^ of the MIM photodetector under TE polarized illumination as a function of bias and frequency.

The current noise spectral density was measured for the MIM photodetector and the reference photodetector for frequencies ranging from 500 Hz to 100 kHz by using a spectrum analyzer (Figure 3a). Both detectors show the same dark currents within 10%. However, the MIM detector shows a slightly higher noise, by about 30%, and this is not yet understood. 1/f noise dominates the low frequency part of the noise spectrum and white noise dominates at the higher frequencies. As the bias is increased from 0.1 to 5 V the white noise as well as the 1/f noise increases, and the 1/f noise becomes increasingly dominant over the whole measured frequency range.

Literature on CQD photoconductors often neglects 1/f noise, or assumes that the white noise is a simple combination of Johnson noise and shot noise as
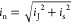
1where  and  where *k*_B_ is the Boltzmann constant, *T* is the temperature, *R* is the resistance of the device, *e* is the elementary charge, and *I* is the dark current. Here, we measured white noise *i*_n_ by fitting the noise spectra. As shown in Figure 3b, the white noise of the MIM photodetector is much smaller than the calculated white noise according to eq 1, but it converges to the Johnson noise at low bias. This is because shot noise is a property of tunnel barriers, while it is well-known that metallic conductors have no shot noise.^30^ Semiconductors however have generation–recombination noise^31^ given as . The generation–recombination noise is due to carrier number fluctuations from thermal generation and recombination.

Thus, the proper white noise expression should be
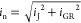
2Since the gain *G* of the device tends to zero at zero bias, *i*_n_ still tends to the Johnson noise for small biases. Using eq 2, the noise-derived photoconductive gain, *G*_noise_, is directly obtained from the measurement of the white noise *i_n_* and the dark current *I*, as . As shown in Figure 3c, *G*_noise_ reaches 0.15 at a 1 V bias.

The responsivity can also be used to obtain a photoconductive gain. The photoconductive gain is defined as  with  the responsivity of the device, *h* the Planck constant, *c* the velocity of light, and  the proportion of photons absorbed in the CQD film. For this definition to be physically meaningful, all of the absorbed photons in the CQD film must generate photocarriers. In a homogeneous system, the value of *G*_ph_ should not depend on the choice of  nor on the choice of polarization. We use the TE polarized illumination, the simulated absorption , and the measured  to get *G*_ph_ as a function of the applied bias (Figure 3c). *G*_ph_ is larger than *G*_noise_ by a factor of ∼2 but it is consistent, by design, with the measured responsivity and absorbed flux.

The 2-fold discrepancy between *G*_noise_ and *G*_ph_ is not yet understood. It may arise from experimental errors, but it may also be due to the inhomogeneous optical and electric field distributions. In particular, we do not have a good assessment of whether generation and recombination rates may be affected by strong Purcell effects near the antennas. This will be a topic of future work.

The 1/f noise of CQD devices has previously been discussed.^32,33^ The conduction has been described by ,^33^ where *S*_i_ is the 1/f noise spectral power density, *A*_i_ is a constant, and *f* is the frequency. The coefficient β was found to be constant and negative for all the CQD samples tested, comprising CdSe, CdSe/CdS, ZnO, and HgTe CQDs.^33^ Our results diverge from that observation (Figure 3d). In our case the coefficient β is found to vary with *I* such that its switches from negative  for small dark currents to positive () for larger dark currents. The magnitude of the 1/f noise can be quantified with the unitless number , with *N*_NC_ the number of quantum dots in the device. Assuming random close packing of the CQD and a quantum dot diameter of 10 nm, we obtain values of α_NC_ (Figure 3d) that are roughly 1 order of magnitude lower than the one previously reported for HgTe CQDs.^33^ This reduced magnitude of 1/f noise can be explained by the higher conductivity of our CQD film associated with higher interdot conductance. This is supported by the comparison with previously reported values of α_NC_ for less conductive films of CQDs^33^ (Figure S7).

The spectral specific detectivity was directly calculated from the spectral responsivity , the measured current noise *i*_N_, and the device area *A* such that . Figure 3e shows the spectral detectivity of the MIM photodetector under 0.75 V bias for both TE and TM polarized illumination and for noise measured at 80 kHz. The absorption enhancement provided by the MIM structure results in a large detectivity improvement at the resonant wavelength with a maximum detectivity  Jones. At the same wavelength and under the same conditions, the reference detector features a detectivity  Jones, which means that the resonant structure provides a 15-fold enhancement to the detectivity of the MIM device. This enhancement is lower than the 19-fold enhancement of the responsivity because the noise is slightly larger with the MIM photodetector.

Since the 1/f noise increases rapidly with bias at low frequencies, the applied bias can be optimized for a particular frequency, as shown in Figure 3f. At 500 Hz, the maximum detectivity is  Jones at 0.2 V bias. At 12 kHz, the maximum detectivity is  Jones at 0.5 V bias. As the best detectivity values are obtained at high frequencies, it is important to ensure that the MIM photodetector is fast enough to respond at high frequency inputs. This is demonstrated by measuring the photoresponse of the photodetector under illumination by a pulsed laser diode (Figure S8). These detectivity values were obtained consistently with several photodetectors (Section S9). Over four different MIM devices, the averaged peak detectivity obtained under 0.75 V bias and at 80 kHz is  Jones.

### Photoluminescence Enhancement from the Nanoantenna Substrates

**Figure 4:**
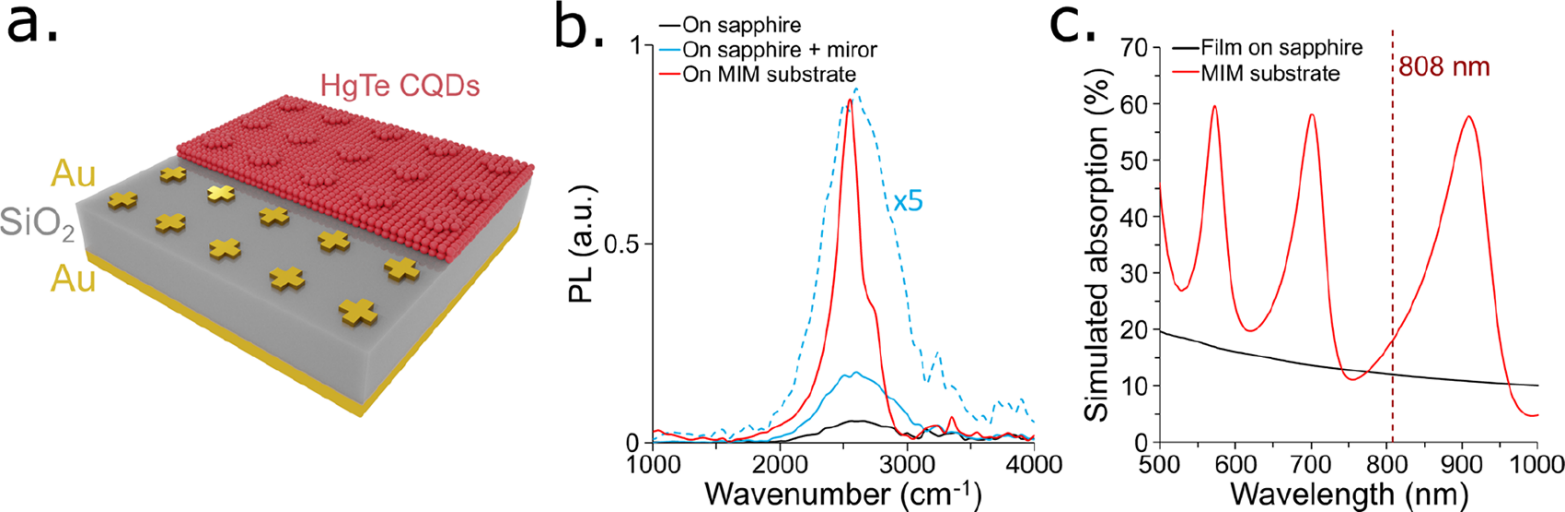
(a) Schematic of the MIM structure for PL measurements. (b) Measured PL intensity of a HgTe CQD film deposited on top of the MIM structure (red), on top of the sapphire substrate (gray), and on top of the sapphire substrate with a gold mirror placed at the back of the substrate (light blue). The dashed curve is the same as the light blue curve multiplied by 5 to visualize the shape of the spectrum. (c) Simulated absorption in the CQD layer of the MIM structure for visible light. The dashed red line marks the wavelength of the pump laser used for the PL experiments.

In addition to improving detector performance, the MIM resonant structure is also expected to enhance the emission properties of the CQD layer by increasing the light–matter coupling around the nanoantennas. Kirchhoff’s law states that the thermal emission of a material *I*(λ) is directly linked to its absorption *A*(λ) by the relation  where  is Planck’s law and *T* is the equilibrium temperature. This relation has been extended to the case of semiconductors where the temperature is that of electrically or optically injected carriers in the material.^34,35^ This has been used to control the emission properties of a material by tailoring its absorption cross-section using optically resonant structures.^36^ To look for an emission enhancement from the MIM structure, we fabricated an identical MIM structure without interdigitated electrodes (Figure 4a). On the same sapphire substrate, a large area was kept free from any structure in order to serve as a PL reference. HgTe CQDs were then deposited on the substrate by spin-coating, and the PL was measured using a FTIR setup and a pump laser at 808 nm. Figure 4b shows the measured PL spectrum for CQDs on the MIM structure and on the bare sapphire substrate. As can be seen, the MIM structure provides a strong improvement to the PL at 2555 cm^–1^ with a 16-fold increase in the PL intensity in comparison with the CQD layer on the sapphire substrate. The resonance peak is slightly shifted in comparison with the MIM photodetector discussed above because of the absence of electrodes in the periodic array of nanoantennas. The CQD film deposited on the substrate used for PL measurement is also slightly thinner (∼70 nm) than the film deposited on the photodetector substrate (∼80 nm) (see Figure S10 for the thickness measurement). The PL signal obtained with the CQD film deposited on sapphire is quite weak and the spectrum is noisy, so we also measured the PL signal of the same film but with a gold mirror on the bottom face of the sapphire substrate. This effectively doubles the pump power and the PL collection resulting in a stronger PL signal while preserving the spectral line shape. The resulting unenhanced PL spectrum can be more easily compared to the MIM enchanced PL, which is substantially narrowed. The PL signal enhancement provided by the MIM structure may also partially originate from the enhancement of the pump absorption in the CQD film as a result of the optical interferences generated by the back reflector and SiO_2_ layer. This point is addressed by the simulated optical absorption of the structure in the visible range, showing a possible limited enhancement by a factor 1.5 to the absorption at 808 nm (Figure 4c).

The enhancement is, therefore, mostly an emission enhancement. It becomes striking considering that the 808 nm pump absorption is weakly affected by the antennas, while the mid-IR emission is mostly from the very small area that is strongly coupled. This suggests a very significant local PL emission efficiency enhancement. Assuming the light is emitted from 1% of the area, the enhancement must then be about 1000-fold for light emitted toward the normal, in qualitative agreement with Kirchoff’s law. Speculating further, the PL emission of the CQDs starts as very weak, with a quantum yield estimated at ∼0.1%. The CQDs may then be very bright near the antennas. Further work will be needed to verify such a large local enhancement experimentally.

There are further interesting consequences for this work. One of those is that a photoconductor structure that could limit the dark current collection to enhanced areas should provide enormous benefits to the detectivity. Possibly, by reaching radiative limited Purcell enhanced emission, the detectors will be close to background limited at room temperature.

Another interesting point is that the CQD films benefit from an enhanced emission despite being deposited directly in contact with the metallic structures, which should enhance nonradiative relaxation and compete with radiative enhancement. Enhanced PL has been previously observed with PbS CQDs directly deposited on square metal antenna arrays.^36^ This is in contrast to the common observation of quenching on metal surfaces.^37^ Here, we still observe enhanced PL even with very thin films of HgTe CQDs (∼25 nm) (Figure S11). This is the benefit of the spatial extent of the electromagnetic field associated with the antenna resonances, where CQDs can be within the enhancement area, yet far enough from the metal.

## Conclusion

We presented the design of a HgTe CQD photoconductor featuring an MIM resonant structure for room temperature photodetection in the mid-IR. The MIM architecture features a strong optical resonance at 2650 cm^–1^, providing a substantially enhanced absorption within a narrow spectral band for a very thin layer of CQDs. This was confirmed by nanofabrication and measurements. The MIM photodetector showed a 19-fold increase in the responsivity and a 15-fold increase in the detectivity at the resonant energy.

The noise of the devices was analyzed in terms of the 1/f noise and white noise. The white noise is found to be smaller than the shot noise, a possibility that is known in photoconductors but often overlooked in the literature on nontraditional 2D or nanocrystal infrared detector materials. Instead, the white noise provides a determination of the photoconductive gain, which is independent from a responsivity determination.

A peak detectivity of 9 × 10^9^ Jones at 2650 cm^–1^ was obtained at 80 kHz. This was achieved with an ultrathin layer of only 80 nm of CQDs. This detectivity is about 40 times better than a standard pyroelectric detector at sub 100 Hz bandwidth. Therefore, a potential application of such fast, sensitive, and spectrally selective mid-infrared CQD detectors could be improved low-cost gas sensing technology.

With the absorption in the ultrathin CQD layer already reaching 60% according to simulations, future improvements will take greater advantage of the strong confinement of the electromagnetic field close to the metallic nanoantennas. Trying to match the dark current collection and the optically enhanced areas should preserve the responsivity and reduce the noise, and this should already boost the detectivity. Furthermore, the most fundamental limiting factor for the mid-infrared CQD detector performance is the low CQD emission efficiency. This work shows that the MIM structure already enhances the spatially averaged photoluminescence emission by 16-fold at resonance energy. This indicates a much greater enhancement of the radiative recombination around the nanostructure since the optical pump excitation is not locally enhanced by the MIM resonant structure. The enhanced brightness also shows that the nonradiative processes are not as enhanced by the proximity of the metal structures. Thus, the improved emission efficiency around the MIM structure described here is another very promising avenue to explore for photodetectors.

## Methods

### Chemicals

Mercury(II) chloride (HgCl_2_, Sigma-Aldrich, ≥99.5%, catalogue no. 215465, 100 g), oleylamine (Sigma-Aldrich, catalogue no. 909831, 500 g), 1,2-ethanedithiol (EDT, Sigma-Aldrich, ≥98.0%, catalogue no. 02390, 100 mL), trioctylphosphine (Sigma-Aldrich, 90%, catalogue no. 117854, 500 mL), 2-mercaptopropionic acid (MPA, Thermo Scientific, 97%, catalogue no. L10257.22, 100 g), hydrochloric acid (Sigma-Aldrich, 37%, catalogue no. 320331, 2.5 l), sulfur powder (Sigma-Aldrich, catalogue no. 215236, 500 g), octylamine (Sigma-Aldrich, 99%, catalogue no. 05802, 500 g), tellurium granules (Sigma-Aldrich, 99.99%, catalogue no. 263303, 25 g), chlorobenzene (Sigma-Aldrich, ≥99.5%, catalogue no. 23570, 2.5 L), tetrachloroethylene (TCE, Sigma-Aldrich, ≥99.0%, catalogue no. 443786, 2.5 L), 1-methyl-2-pyrrolidinone (NMP, Sigma-Aldrich, ≥99.0%, catalogue no. 443778, 2.5 L), 2-propanol (IPA, Fisher, catalogue no. A451-4), acetone (Fisher, catalogue no. A18-4), and amyl acetate (Sigma-Aldrich, ≥99%, catalogue no. W504009, 1 kg).

#### HgTe CQDs Synthesis

HgTe CQDs were prepared according to the literature.^6^ First, 86 mg of HgCl_2_ were added to a 20 mL glass vial containing 5 mL of degassed oleylamine. The mixture was then placed in a glovebox under a nitrogen atmosphere and heated to 110 °C with stirring for 30 min to dissolve the HgCl_2_. A nucleation step is achieved by quickly injecting a premixed solution of 80 μL of TopTe, 30 μL of DDT, and 300 μL of oleylamine and letting the reaction progress for 1 min. The CQDs are then grown by adding a solution of 150 μL of TopTe and 300 μL of oleylamine drop by drop over 4 min and then letting the reaction progress for 2 more minutes. The reaction is then quenched by quickly injecting 5 mL of tetrachloroethylene (TCE) and letting the mixture cool at room temperature for 4 min. Finally, 200 μL of mercaptopropionic acid (MPA) were added and the CQD solution was stirred at room temperature for 2 h.

The CQD solution was finally washed by precipitation in IPA and redispersed in a solution of 5 mL of chlorobenzene (CBZ), 200 μL of TOP, and 60 μL of DDT.

Before spin-coating on a substrate, 200 μL of the CQD solution was washed a second time by precipitation in IPA and redispersed in 60 μL of CBZ.

#### Finite Element Simulations

The optical simulations were conducted by using COMSOL Multiphysics in the frequency domain with the RF module. The structures were simulated with a 3D model featuring Floquet periodic conditions on the side boundaries of the system. The incident electromagnetic waves were introduced through a periodic excitation port at the top boundary. On the other hand, the bottom boundary was defined as a perfect electric conductor for simulations of the MIM structure and as a listening port for simulations of the reference structure. The region on top of the CQD film was defined as air with a refractive index . Refractive indexes were extracted from the literature for SiO_2_,^38^ gold,^39^ and sapphire.^38^ For the HgTe CQD film, the real part of the refractive index was set to a constant value , close to previously reported values of the refractive index of similar HgTe CQD films.^40,41^ The spectral profile of the extinction coefficient was extracted from the responsivity measurements, and its amplitude was normalized so that its value is 0.1 at the top of the first exciton peak.

#### Fabrication of Photodetector Substrates

The fabrication starts with a 2 in. C-plane sapphire wafer. After being rinsed with acetone and isopropanol, the wafer is coated with a layer of AZ MIR 703 (19cPs) photoresist by spin-coating at 3500 rpm for 45 s followed by a 1 min bake at 95 °C. Using a Heidelberg MLA150 direct write lithographer equipped with a 375 nm laser, the resist layer is exposed with a dose of 120 mJ/cm^2^ to pattern a set of 800 × 800 μm squares. The wafer is then baked at 115 °C for 1 min, and the resist is developed in AZ 300 MIF for 1 min before rinsing with water. A stack of 5 nm of titanium, 100 nm of gold, and 0.5 nm of titanium is deposited on the surface of the wafer with an Angstrom EvoVac electron beam evaporator. After conducting a lift-off process by sonicating the wafer in *N*-methyl-2-pyrrolidone (NMP) at 80 °C for 10 min and rinsing its surface with NMP, acetone, and IPA, we obtain a set of square gold mirrors that will serve as the back reflectors of the MIM photodetectors.

A 1 μm thick SiO_2_ layer is then grown on the entire surface of the wafer using a Plasma-Therm Apex SLR HDPCVD.

This is followed by a second photolithography step to pattern macroscopic pads that serve as electrical contacts for the electrodes of the devices. This lithography step also relies on the AZ MIR 703 photoresist and Heidelberg MLA150 and uses the same parameters mentioned above. After exposure and development of the resist, the wafer is placed in the Angstrom EvoVac evaporator to deposit 2 nm of titanium and 50 nm of gold. After a lift-off process in NMP, the architecture features large deported electrical contacts but still lacks electrodes.

The interdigitated electrodes are patterned through e-beam lithography. This is achieved by spin-coating the wafer with a layer of AR-P 6200-09 at 4000 rpm for 45 s. The wafer is then baked at 150 °C for 1 min. A conductive top layer is also added by spin-coating the wafer with AR-PC 5090.02 at 2000 rpm for 45 s followed by a baking at 90 °C for 2 min. The interdigited electrodes and nanoantennas of the MIM photodetector as well as identical interdigited electrodes for the reference photodetectors are patterned during the same e-beam lithography with a Raith EBPG5200 at 100 kV. The resist is exposed with a dose of 450 μC/cm^2^ with the e-beam current set to 10 nA. An alignment procedure ensures that the electrodes and nanoantennas of the MIM photodetectors are patterned above the gold back reflectors previously fabricated. Conversely, the electrodes of the reference photodetectors are patterned in areas of the wafer lacking back reflectors. After the e-beam exposure, the wafer is rinsed with water to remove the conductive top layer of AR-PC 5090.02. The e-beam resist is then developed for 1 min in amyl acetate and rinsed in IPA. Using the Angstrom EvoVac evaporator, 1 nm of titanium and 50 nm of gold are deposited. The wafer is then sonicated for 10 min at 80 °C in NMP and rinsed with NMP, acetone, and IPA.

Finally, the wafer is diced with a Disco DAD3240 automatic dicing saw to obtain nine identical 12 × 12 mm square substrates. Each of these substrates features a set of four MIM photodetectors and two reference photodetectors packed in a small central area and with large contact pads on the sides of the substrate.

To complete the fabrication of a photodetector substrate, two layers of HgTe CQDs are spin-coated on the substrate at 1500 rpm. Each layer is cross-linked with a 1:1:100 solution of EDT, hydrochloric acid, and IPA. After the deposition of the two layers, the film is treated with a solution of sulfur ions in IPA and baked at 60 °C for 1 h.

#### Fabrication of PL Substrates

The substrate used for PL measurements is fabricated following steps similar to those for the photodetector devices. The fabrication starts with a 2 in. C-plane sapphire wafer. Large square back reflectors with surface areas of 3 × 3 mm are patterned using the AZ MIR 703 photoresist and the Heidelberg MLA150. The back reflectors are obtained by the deposition of 5 nm of titanium, 100 nm of gold, and 0.5 nm of titanium with the Angstrom EvoVac evaporator followed by a lift-off process in NMP.

Before the addition of the SiO_2_ layer, a second photolithography step is performed to pattern 3 × 3 mm squares. For this second lithography, the substrate is spin-coated with AZ nlof 2020 at 4000 rpm for 45 s, baked at 110 °C for 1 min, and exposed with a dose of 210 mJ/cm^2^ using the Heidelberg MLA150. The substrate is then baked at 100 °C for 1 min to perform an image reversal followed by a 1 min development in AZ 300 MIF before rinsing with water. Residual exposed photoresist is removed with a O_2_ plasma exposure at 200 W and 60 sccm O_2_ for 75 s in a YES-CV200. A 1 μm thick SiO_2_ layer is then grown on the surface of the wafer using the Plasma-Therm Apex SLR HDPCVD. After a lift-off process in NMP, the SiO_2_ layer covers the entire surface of the wafer except for the 3 × 3 mm squares patterned during the second photolithography where the sapphire substrate is bare.

Arrays of nanoantennas are patterned above the fabricated back reflectors through e-beam lithography with the AR-P 6200-09 and AR-PC 5090.02 resists and using the Raith EBPG5200. All of the parameters used during this step are the same as the ones used for the e-beam lithography step conducted during the fabrication of photodetectors. After the development of the resist, 1 nm of titanium and 50 nm of gold are evaporated on the wafer by using the Angstrom EvoVac evaporator. The wafer is then sonicated for 10 min at 80 °C in NMP and rinsed with NMP, acetone, and IPA.

Finally, the wafer is diced with the Disco DAD3240 automatic dicing saw to obtain nine identical 12 × 12 mm square substrates. Each of these substrates feature a set of three MIM structures each covering a surface of 3 × 3 mm as well as a 3 × 3 mm area where the sapphire substrate is bare.

To complete the fabrication of a PL substrate, two layers of HgTe CQDs are spin-coated on the substrate at 1500 rpm. Each layer is cross-linked with a 1:1:10 solution of EDT, hydrochloric acid, and IPA. After the deposition of the two layers, the film is treated with a solution of sulfur ions in octylamine and baked at 60 °C for 1 h.

#### Photodetector Characterization

The photodetectors were placed in a cryostat but characterized under a vacuum at room temperature. The device was illuminated with an Omega BB-4A blackbody calibrator at 600 °C with its emission chopped at 100 Hz. The signal was measured with a Femto DLPCA-200 low-noise transimpedance amplifier, a Stanford Research Systems SR560 Low-Noise Preamplifier, and an oscilloscope. The noise spectral density of the device was measured with a Stanford Research Systems SR760 FFR spectrum analyzer. The photoresponse spectrum of the device was measured with a Nicolet Magna IR 550 FTIR and normalized with the photo response of the internal DTGS detector.

The photoresponse spectra  are measured directly from the FTIR using the samples as external photodetectors with the light focused by a parabolic gold-coated mirror with F/5 optics. The spectra, which include the effect of the spectrometer blackbody source and the optics, are then normalized to the spectrum of the internal DTGS detector, , and divided by the frequency , such as, . This provides a relative responsivity spectrum, which accounts for the fact that the DTGS detector is a slow pyroelectric detector, while the samples are fast detectors. The absolute spectral responsivity is then obtained by the separate measurement of the photocurrent *I*_s_ under illumination by a blackbody source at 873 K. The incident power per cm^–1^ is calculated given the 21 mm diameter source, placed 15 cm from the sample, and accounted for 10% losses through the sample holder CaF_2_ window. The absolute responsivity is given by . The polarized absolute responsivities *R*_TE_ and *R*_TM_ are similarly obtained using *I*_s_ and relative responsivities *R*_rel,TE_ and *R*_rel,TM_ measured with polarized illumination in the FTIR setup such that .

#### PL Measurements

The PL spectra were recorded by using a step-scan FTIR spectrometer and a cooled MCT detector. The substrate was illuminated with an 808 nm laser diode modulated at 40 kHz, and the PL signal was collected by a F/2 gold mirror and sent through the FTIR setup. A silicon wafer was placed in front of the MCT detector to filter out the pump laser. The output of the MCT detector was sent through a lock-in amplifier. The recorded spectra were normalized by the transfer function of the setup and calibrated with the thermal emission of a tungsten filament at 1293 K.

## Supporting Information Available

Optical losses resulting from the spectral alignment of the λ/4 resonance and nanoantenna array resonance; simulated absorption at non-normal incidence; absorption enhancement from the optimized λ/4 substrate; absorption enhancement in thick films of CQDs; *I*–*V* curve of the MIM photodetector; polarization dependence of the reference photodetector; comparison of the 1/f noise with previously reported results; response time of the photodetectors; performance reproducibility; thickness of the CQD film deposited on the PL substrate; and PL enhancement of thin films (PDF)
