# Supplementary material for: Uncooled High Detectivity Mid-Infrared Photoconductor Using HgTe Quantum Dots and Nanoantennas
Source: ACS Nano. 2024 Mar 11;18(12):8952–60. doi: 10.1021/acsnano.3c12581 (PMC10976962; doi:10.1021/acsnano.3c12581)
Supplement: Supplementary file 1 — nn3c12581_si_001.pdf [file nn3c12581_si_001.pdf]

Supporting information

Uncooled high detectivity Mid-Infrared photoconductor using HgTe quantum dots and nanoantennas.

Augustin Caillas, Philippe Guyot-Sionnest

James Franck Institute, 929 East 57th Street, The University of Chicago, Chicago, IL 60637, USA.

pgs@uchicago.edu

Supporting information:

1. Optical losses resulting from the spectral alignment of the λ/4 resonance and nano-antenna array resonance
2. Simulated absorption at non-normal incidence
3. Absorption enhancement from the optimized λ/4 substrate
4. Absorption enhancement in thick films of CQDs
5. I-V curve of the MIM photodetector
6. Polarization dependance of the reference photodetector
7. Comparison of the 1/f noise with previously reported results
8. Response time of the photodetectors
9. Performance reproducibility
10. Thickness of the CQD film deposited on the PL substrate
11. PL enhancement of thin films

1. Optical losses resulting from the spectral alignment of the $\lambda/4$ resonance and nanoantenna array resonance

From simulation results, we found that the absorption in the CQD film of the MIM structure is maximized if the resonance of the nanoantenna array and the resonance of the λ/4 layer are spectrally displaced from each other. In the optimized structure the λ/4 layer is composed of the 1000 nm thick SiO_2 layer together with the 80 nm thick HgTe CQD film, on top of the gold back reflector. It produces a broad λ/4 resonance peaked at 1600 cm^{-1} while the nanoantenna array produces a resonance at 2650 cm^{-1}. In this case, the absorption in the 80 nm CQD film reaches 60% for TE polarized illumination. Reducing the SiO_2 thickness to 700 nm in order for the λ/4 resonance to peak at 2650 cm^{-1} reduces the CQD film absorption to 45% while the optical losses in the metallic structures increases (figure S3).

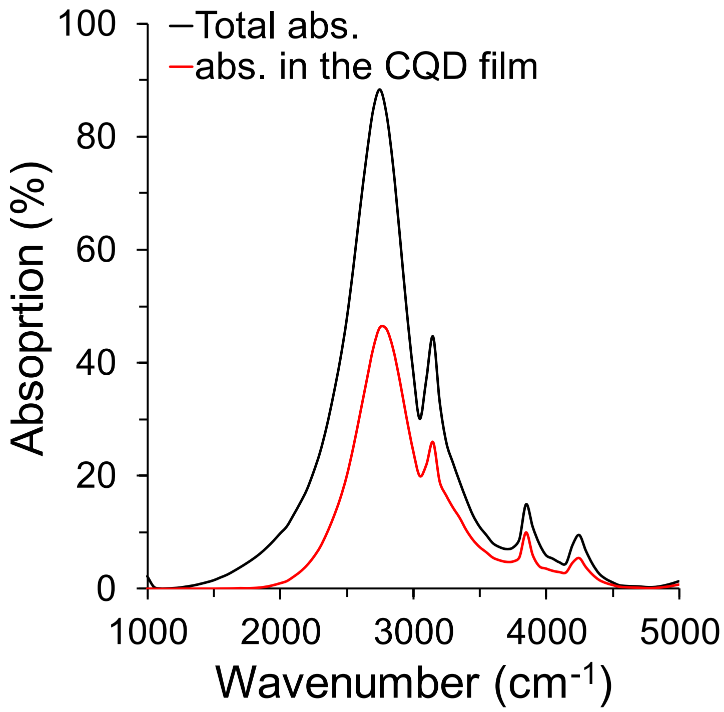

Figure S1: Simulated absorption for a MIM structure featuring a 700 nm thick SiO2 layer, with λ/4 resonance at the same energy than the nanoantenna array (2650 cm-1). The peak optical absorption in the CQD layer is 45%, which is lower than the 60% absorption obtained if the λ/4 resonance is shifted to 1600 cm-1.

2. Simulated absorption at non-normal incidence

Additional simulations were conducted to study the incidence angle dependance of the absorption of the MIM structure.

In the simulations, the angle between the normal to the surface of the structure and the wavevector of the incident electromagnetic field was varied between 0° and 20° by increments of 2.5°. This was performed for both polarization and for both orientation of the plane of the incidence (perpendicular to the electrodes and parallel to the electrodes). Finally, the spectra simulated in a range of angles are averaged to obtain the absorption spectrum under illumination with corresponding F-number optics. The averaging is weighted with the sinus of the angle to take into account the fact that the amount of light focused by the optics on the structure increases with the angle of incidence.

With our notation, the TE polarization corresponds to an electric field polarized along the electrodes and the TM polarization corresponds to an electric field polarized perpendicular to the electrodes, independently of the direction of the plane of incidence.

Under illumination with F/5 optics the absorption of TE polarized light reaches 57% at the peak of the resonance which is close to the 60% obtained at normal incidence. This demonstrates the robustness of the TE polarized resonance with illumination at non-normal incidence. On the other hand, the absorption of TM polarized light is reduced to 33% which is a 1.3-fold reduction compared to the absorption at normal incidence.

Under illumination with a F/1.4 optics the absorption for TE and TM polarized light is further reduced which shows that the best photodetection performances should be obtained by using large F-number optics.

The unpolarized absorption spectra can also be calculated by averaging the absorption for both polarizations. While the absorption is reduced with illumination at larger angles of incidence, the shape of the unpolarized spectrum remains relatively similar with illumination with F/1.4 and F/5 optics.

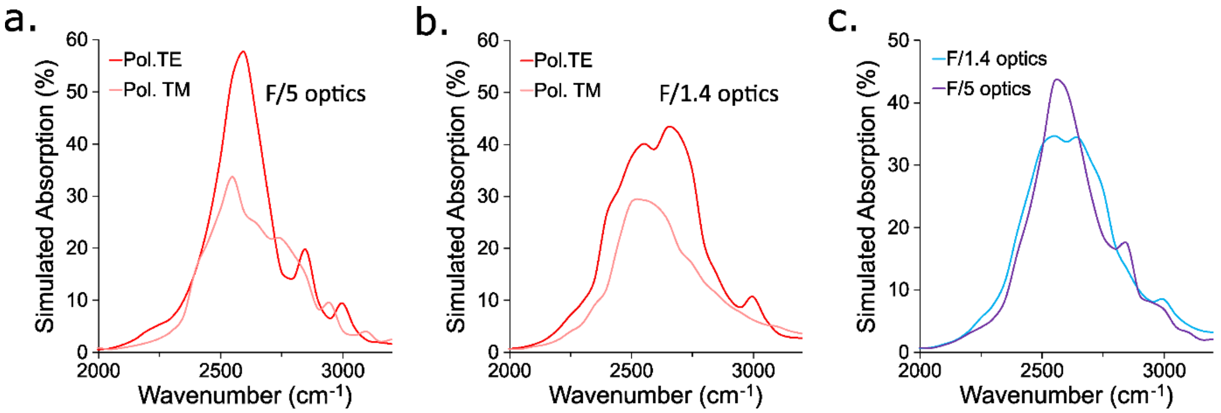

Figure S2: a) Simulated absorption in the 100 nm HgTe CQD film of the MIM structure for TE and TM polarized illumination with F/5 optics. b) Simulated absorption in the 100 nm HgTe CQD film of the MIM structure for TE and TM polarized illumination with F/1.4 optics. a) Simulated absorption in the 100nm HgTe CQD film of the MIM structure for unpolarized illumination with F/1.4 and F/5 optics.

3. Absorption enhancement from the optimized $\lambda/4$ substrate

To stress the benefit of including plasmonic nanoantennas in the design of the MIM photodetector, we conducted additional COMSOL simulations with a λ/4 structure optimized to maximize the absorption at 2650 cm^{-1}. The structure simply consists of an 80 nm thick HgTe CQD film on top of a 700 nm thick SiO_2 layer and back reflector.

The λ/4 resonance generates a broad resonance peak with a 5-fold enhancement to the absorption at 2650 cm^{-1} compared to the same 80 nm thick film deposited on top of a sapphire substrate. While being simpler than the MIM structure, this architecture displays a much lower potential for the improvement of the performances of CQD optoelectronics.

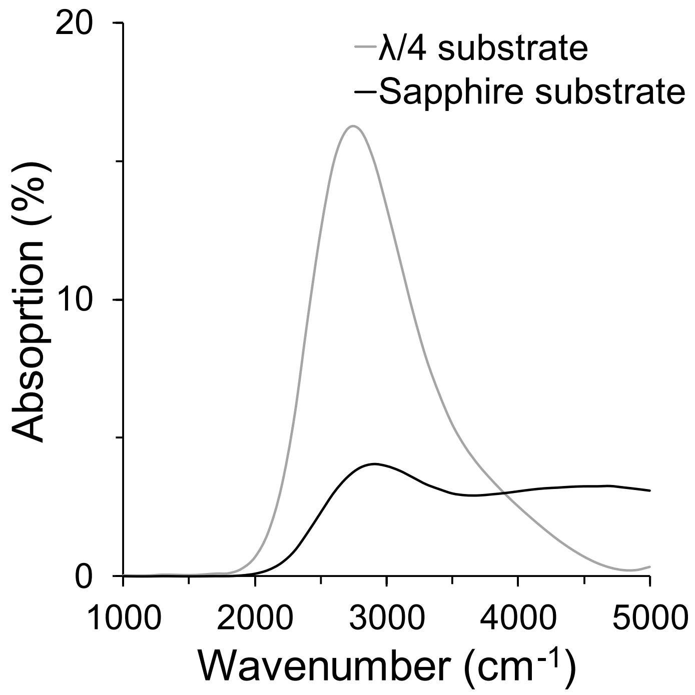

Figure S3: Simulated enhancement of the absorption of a 100 nm HgTe film on a λ/4 layer at 2650 cm^{-1}. The absorption of the film on the λ/4 layer is compared to the absorption of an identical film on a sapphire substrate.

4. Absorption enhancement of thick CQD film with the MIM structure

The boost to the absorption of the CQD film from the MIM structure is maximized for thin CQD films. This is demonstrated by simulating the absorption in the CQD film for several film thickness (Figure S4). In these simulations, the thickness of the film was increased while the thickness of the SiO2 layer was decreased accordingly in order to maintain the λ/4 resonance at the same energy (1600 cm-1). One could expect that thicker films should provide larger absorption. However, increasing the CQD film thickness actually reduces the absorption because as the film becomes thicker, the MIM resonance becomes weaker. This is explained by the fact that the λ/4 resonance produces constructive interferences at the top of the λ/4 layer, which is the top of the CQD film. However, the antennas are placed at the bottom of the CQD film. Then, increasing the CQD film thickness spatially displace the λ/4 constructive interferences from the antenna array, resulting in a weaker MIM resonance. As a result, as the CQD film thickness is increased the resonance absorption peak becomes weaker and broader. It also redshifts because the shape of the absorption spectrum becomes increasingly dominated by the broad λ/4 resonance rather than the narrow nanoantenna array resonance.

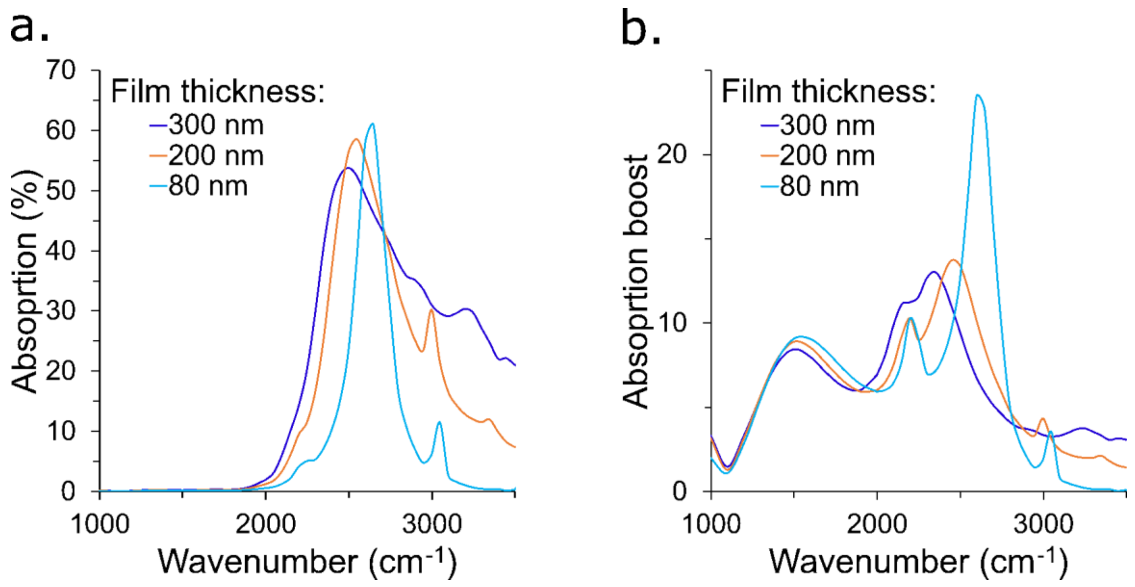

Figure S4: Simulated absorption of the MIM structure for several CQD film. In these simulations, the thickness of the SiO2 layer has been adjusted to maintain the λ/4 resonance at the same energy (1600 cm-1). To calculate the absorption boost, the absorption in the CQD film is compared to the absorption of an identical film on a bare sapphire substrate.

5. I-V curve of the MIM photodetector

The I-V curve of the MIM photodetector displays a linear behavior under background illumination and under illumination from an 873 K blackbody. The photocurrent only contributes for a small part of the total current because we characterize the devices at room temperature and with low illumination power density (0.01 W/cm²) such that the conduction band of the CQD film is populated mainly by thermal carriers rather than by photocarriers.

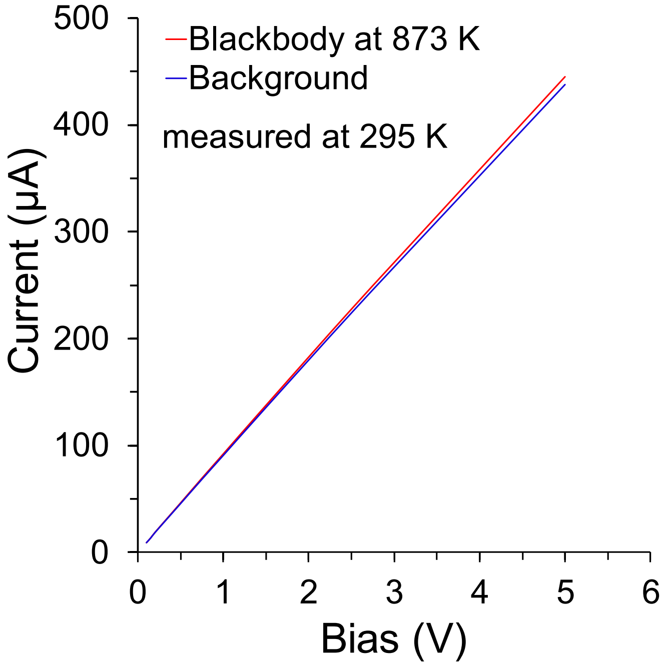

Figure S5: I-V curve of the MIM photodetector measured at room temperature without illumination and under illumination by a blackbody at 873 K.

6. Polarization dependance of the reference photodetector

The responsivity of the reference photodetector displays a polarization dependance which arises from the presence of the interdigited gold electrodes. Because of their periodicity (8.9 µm) the electrodes act as a diffraction grating with a larger diffraction efficiency for TM polarization than for TE polarization. This diffracted light is effectively lost and thus we observe lower responsivity in TM polarization. This is consistent with the simulated absorption of the reference detector which displays the same polarization dependance.

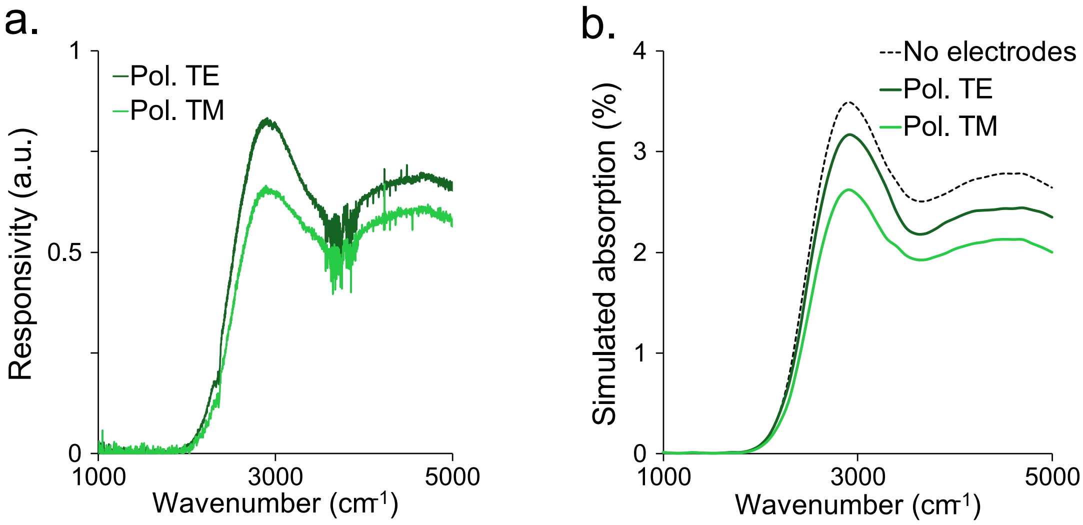

Figure S6: a) Responsivity of the reference photodetector illuminated with TE and TM polarized light. b) Simulated absorption in the HgTe CQD film of the reference photodetector under TE and TM polarized illumination at normal incidence. The dashed line represents the simulated absorption of the same film deposited on a sapphire substrate without electrodes.

7. Comparison of the 1/f noise with previously reported results

Liu et al.[1] proposed an empirical form of the 1/f noise of any CQD solid $\frac{S_i}{I^2} = \frac{\alpha_{NC}}{N_{NC} f}$ where $\alpha_{NC} = 2 \times 10^{-6} \frac{G_0}{G_{NC}}$. S_i is the 1/f noise spectral power density, I is the dark current, N_{NC} is the number of CQDs in the device, f is the frequency, G_0 is the quantum conductance and G_{NC} is the interdot conductance. G_{NC} can be calculated directly from the conductivity of the CQD film σ and the center-to-center distance between QDs d, such that $G_{NC} = \sigma d$. For the CQD films of the MIM photodetector and reference photodetector presented in this work we assumed $d = 10\ nm$ from the QDs size and ligands length. Plotting α_{NC} vs. G_{NC}/G_0 we observe a good agreement with the proposed empirical form of the 1/f noise proposed by Liu et al.

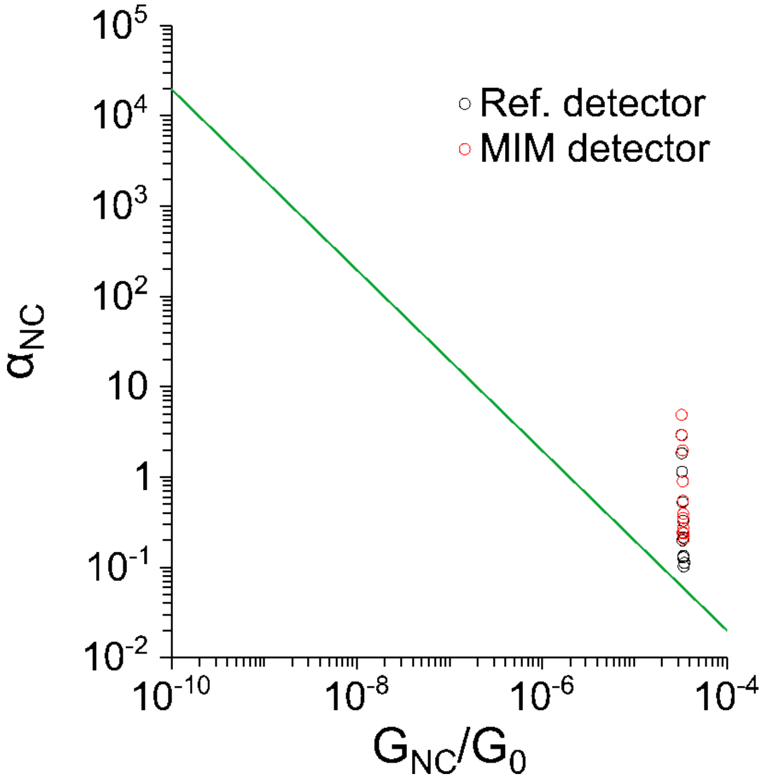

Figure S7: Comparison of the 1/f noise of the MIM and reference photodetectors with previously reported values of the 1/f noise in HgTe CQD film of varying conductivity. The solid green line is the empirical law proposed by Liu et al. for any CQD solid.

8. Response time of the photodetectors

The fast and sensitive response of the MIM photodetector is demonstrated by its interferogram at the fastest scan speed as well as the photoresponse under illumination by a Highland Technology T165 laser diode pulser. The pulse response is about 60 ns for rise and fall, demonstrating MHz operability. Interestingly, both the MIM and reference photodetectors displays identical rise and fall time which shows that the presence of the nano-antennas in the CQD channels of the device have no effect on the electrical transport.

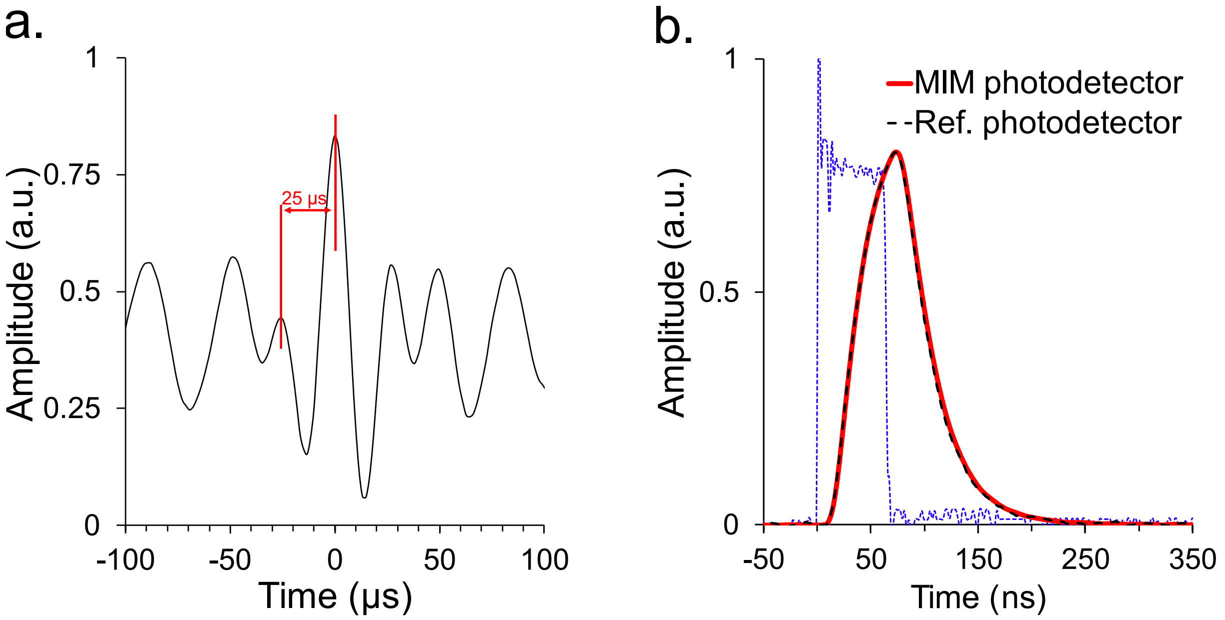

Figure S8: a) Interferogram of the MIM photodetector under illumination by the FTIR at maximum scan speed. b) Photoresponse of the MIM photodetector (red) and reference photodetector (dashed black) for a shorter laser pulse. The electrical pulse driving the pulsed laser is represented by the dashed blue curve.

9. Performance reproducibility

Several MIM and reference photodetectors were characterized to assess performance reproducibility. Table S1 presents the performances of MIM photodetectors and reference photodetectors fabricated on two different substrates and following the same process. Both substrates feature 2 MIM photodetectors and 2 reference photodetectors. The values for responsivity R and specific detectivity D^* correspond to peak spectral responsivity and peak spectral specific detectivity (at 2650 cm^{-1}) under TE polarized illumination. The noise current density i and the specific detectivity D^* are quoted at 500 Hz, 12 kHz and 80 kHz. All the values were obtained under 0.75 V bias.

The normalized responsivity spectra of the 4 MIM photodetectors shows good reproducibility, with very similar shape and resonance energy (Figure S7).

Table S1: Performances of several MIM and reference photodetectors

	**Substrate 1**				**Substrate 2**			
	MIM 1	MIM 2	Ref. 1	Ref. 2	MIM 3	MIM 4	Ref. 3	Ref. 4
$R\ (A/W)$	0.43	0.39	0.02	0.02	0.38	0.40	0.02	0.02
$i_{500Hz}\ (pA/\sqrt{Hz})$	13.8	12.8	10.3	10.8	10.6	15.3	10.5	9.2
$i_{12kHz}\ (pA/\sqrt{Hz})$	2.9	2.8	2.1	2.1	2.4	3.1	2.4	2.0
$i_{80kHz}\ (pA/\sqrt{Hz})$	2.2	2.2	1.7	1.8	2.1	2.6	2.0	1.7
$D^*_{500Hz}(10^9\ Jones)$	1.5	1.4	0.1	0.1	1.7	1.2	0.1	0.1
$D^*_{12kHz}(10^9\ Jones)$	6.9	6.5	0.4	0.4	7.5	6.0	0.3	0.5
$D^*_{80kHz}(10^9\ Jones)$	9.2	8.3	0.6	0.5	8.6	7.2	0.4	0.5

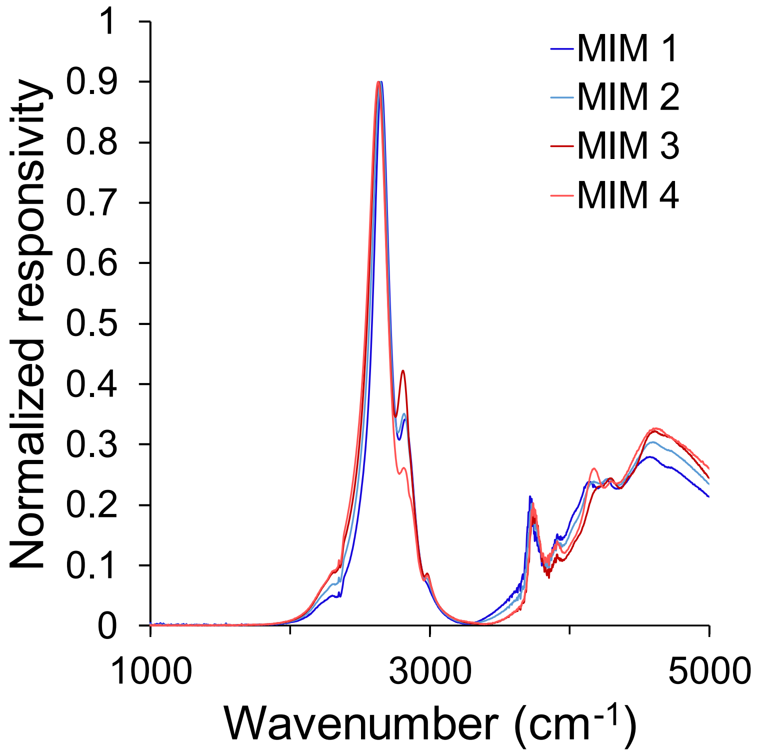

Figure S9: Normalized responsivity spectra of the 4 different MIM photodetector under 0.75 V bias and TE polarized illumination.

10. Thickness of the CQD film deposited on the PL substrate

The thickness of the HgTe CQD film deposited on top of the MIM structure for PL measurements is measured with SEM imaging (Figure S5.a). The substrate is cleaved using a diamond tip and its slice is directly observed. The thickness of the film is 70 nm and is homogenous over the entire MIM structure. The film uniformity extends over the entire substrate as can been observed by looking at the slice of the device a few millimeters further from the MIM structure (Figure S5.b).

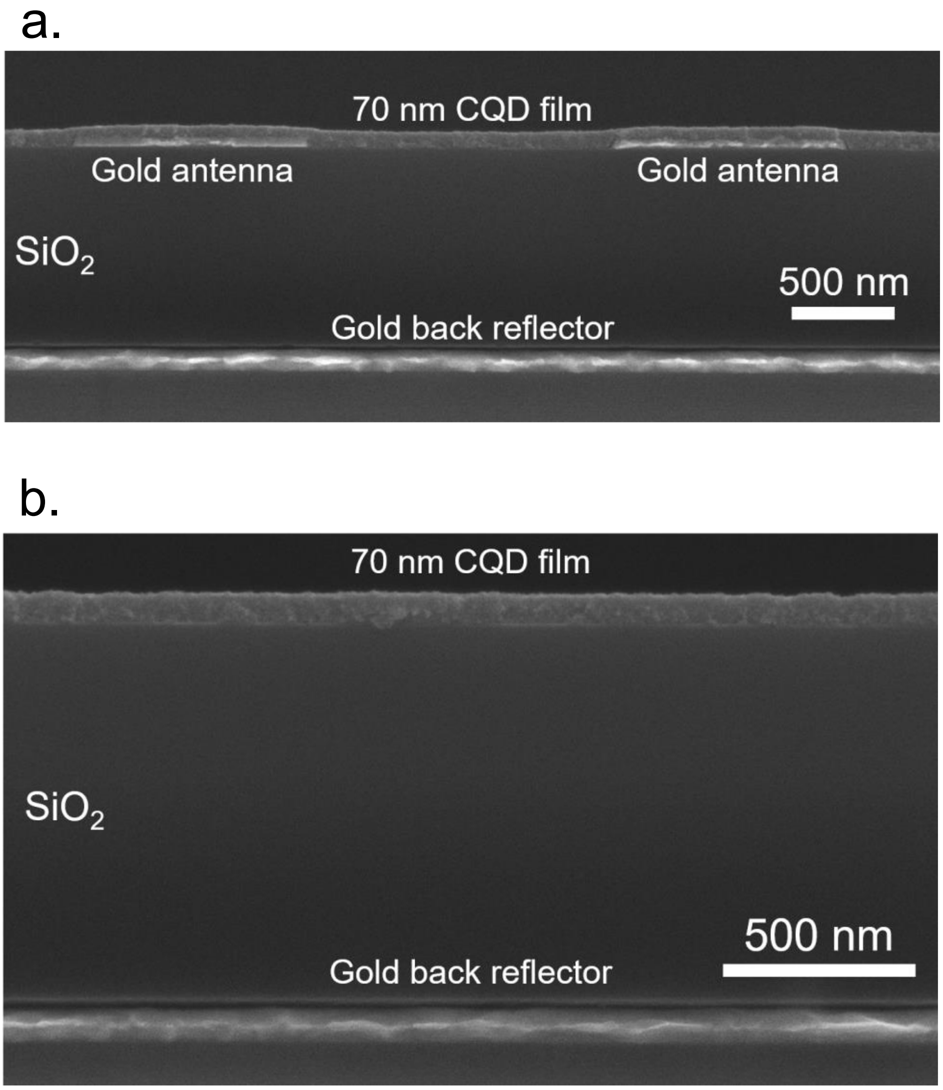

Figure S10: a) SEM observation of the slice of the MIM structure of the PL substrate. b) SEM observation of the slice of the PL substrate a few millimeters away from the MIM structure.

11. PL enhancement of thin films

We conducted additional characterization to demonstrate that the enhancement of the PL of HgTe CQD films is observed even for CQD films with thickness of the order of a few QDs. Here a film of thickness smaller than 30 nm is deposited on the MIM structure by performing two spin-coatings each followed by an EDT treatment. The CQD solution was diluted with chlorobenzene prior to deposition in order to obtain thin layers of CQDs. The thickness of the film (~25 nm) is measured by observing a slice of the structure with a SEM after the PL measurements are done.

The PL of the film on the MIM structure is compared to the PL of the same film on top of the bare sapphire surface, with and without a mirror at the back side of the sapphire substrate. Because the film is very thin the PL signal is weak and the resulting spectra are noisy. With the MIM structure, no quenching of the PL is observed and the enhancement is similar to the enhancement observed with a thicker film.

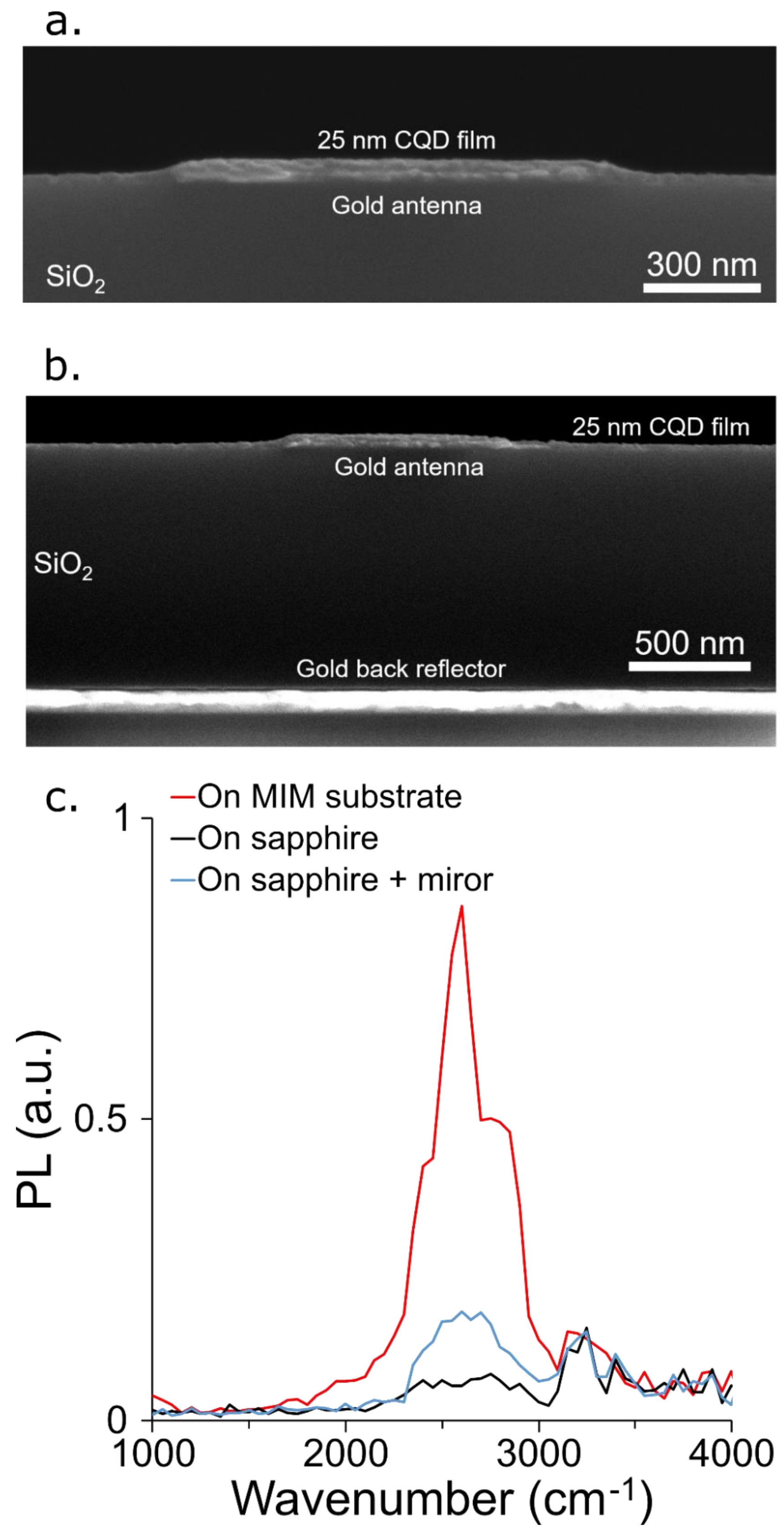

Figure S11: a) SEM observation of the slice of the substrate for PL measurement with a thin film (25 nm). b) Same observation but less zoomed to be able to observe all the elements of the MIM structure. c) PL measurements performed on the corresponding substrate with the MIM structure (red), directly on the sapphire substrate (black) and directly on the sapphire surface with a mirror on the back of the substrate (light blue).

References

(1) Liu, H.; Lhuillier, E.; Guyot-Sionnest, P. 1/f Noise in Semiconductor and Metal Nanocrystal Solids. *Journal of Applied Physics* **2014**, *115* (15), 154309. https://doi.org/10.1063/1.4871682.
